# The impact of diabetes in cognitive impairment: A review of current evidence and prospects for future investigations

**DOI:** 10.1097/MD.0000000000035557

**Published:** 2023-10-27

**Authors:** Nicholas Aderinto, Gbolahan Olatunji, Muili Abdulbasit, Patrick Ashinze, Olamide Faturoti, Abayomi Ajagbe, Bonaventure Ukoaka, Gbolahan Aboderin

**Affiliations:** a Department of Medicine, Ladoke Akintola University of Technology, Ogbomoso, Nigeria; b Department of Medicine and Surgery, University of Ilorin, Kwara State, Nigeria; c Saint Francis Catholic Hospital, Okpara Inland, Warri Catholic Diocesan Hospital Commission, Delta State, Nigeria; d Department of Anatomy, College of Health Sciences, Nile University of Nigeria, Abuja, Nigeria; e Department of Internal Medicine, Asokoro District Hospital, Abuja, Nigeria.

**Keywords:** Alzheimer disease, cognitive impairment, dementia, diabetes mellitus, mild cognitive impairment

## Abstract

Cognitive impairment in individuals with diabetes represents a multifaceted and increasingly prevalent health concern. This review critically examines the current evidence regarding the intricate relationship between diabetes and cognitive decline. It highlights the existing knowledge on the impact of diabetes on cognitive function, spanning from mild cognitive impairment to dementia, including vascular and Alzheimer dementia. The review underscores the need for a standardized diagnostic paradigm and explores research gaps, such as the implications of cognitive impairment in younger populations and various diabetes types. Furthermore, this review emphasizes the relevance of diabetes-related comorbidities, including hypertension and dyslipidemia, in influencing cognitive decline. It advocates for a comprehensive, interdisciplinary approach, integrating insights from neuroscience, endocrinology, and immunology to elucidate the mechanistic underpinnings of diabetes-related cognitive impairment. The second part of this review outlines prospective research directions and opportunities. It advocates for longitudinal studies to understand disease progression better and identifies critical windows of vulnerability. The search for accurate biomarkers and predictive factors is paramount, encompassing genetic and epigenetic considerations. Personalized approaches and tailored interventions are essential in addressing the substantial variability in cognitive outcomes among individuals with diabetes.

## 1. Introduction

In recent years, diabetes mellitus has transitioned from a medical concern primarily associated with metabolic disturbances to a complex condition with potential implications far beyond glycemic control.^[1]^ As the prevalence of diabetes continues to rise globally, a growing body of research has begun to highlight its potential impact on cognitive function.^[2]^ Diabetes, characterized by the body inability to regulate blood glucose levels effectively, is well-known for its associated complications, including cardiovascular disease, neuropathy, and retinopathy.^[3]^ However, a lesser-explored aspect is its potential influence on cognitive abilities. Cognitive impairment, encompassing deficits in memory, attention, language, and executive function, represents a significant challenge to affected individuals, their families, and healthcare providers.^[4]^

The relationship between diabetes and cognitive impairment is multifaceted and intricate.^[5]^ While the exact mechanisms are still unknown, several potential pathways have emerged. Vascular factors, oxidative stress, inflammation, and insulin resistance are among the key players believed to mediate the impact of diabetes on cognitive function.^[6]^ Understanding these mechanisms is crucial, as it can lead to the development of targeted interventions aimed at preserving cognitive health in individuals with diabetes. This review seeks to delve into the current knowledge regarding the interplay between diabetes and cognitive impairment.

## 2. Methodology and study selection

This review employed a literature search to identify pertinent studies investigating the impact of diabetes on cognitive impairment (Table 1). To achieve this, 4 reputable databases, namely PubMed, MEDLINE, Embase, and PsycINFO, were meticulously searched using pertinent keywords and Medical Subject Headings (MeSH) terms. The search was confined to studies published within ten years leading up to August 2023.

**Table 1:** Methodology and study selection.

Step	Procedure
Database selection	Four reputable databases, namely PubMed, MEDLINE, Embase, and PsycINFO, were selected for the literature search.
Literature search	A comprehensive search strategy utilizing relevant keywords and Medical Subject Headings (MeSH) terms was meticulously designed and executed. The search was confined to studies published within the 10-yr period leading up to August 2023.
Inclusion criteria	Inclusion criteria embraced a diverse array of research articles, observational studies, and clinical trials that explored the intricate relationship between diabetes and cognitive impairment. Studies addressing various cognitive domains were eligible for consideration.
Exclusion criteria	Exclusion criteria comprised studies that lacked relevance to the nexus of diabetes and cognitive impairment, studies devoid of pertinent outcome measures, and studies not published in the English language.
Initial screening	Titles and abstracts of identified articles were subjected to meticulous review to assess their relevance to the research topic.
Full-text evaluation	Full-text articles that passed the initial screening were subjected to a comprehensive evaluation in accordance with predefined inclusion and exclusion criteria.
Data extraction	Relevant data from the selected studies were meticulously extracted. This included crucial information such as study design, sample size, key findings, and acknowledged limitations.
Narrative synthesis	Given the inherent heterogeneity in study designs, outcomes, and populations across the selected studies, a narrative synthesis approach was thoughtfully employed. This facilitated the summarization of findings, identification of common themes, emerging trends, and any notable disparities.

### 2.1. Inclusion and exclusion criteria

Inclusion criteria encompassed a spectrum of research articles, observational studies, and clinical trials that delved into the intricate relationship between diabetes and cognitive impairment. Notably, studies addressing various cognitive domains, including but not limited to memory, attention, language, and executive function, were eligible for consideration. Conversely, exclusion criteria encompassed studies lacking relevance to the nexus of diabetes and cognitive impairment, studies bereft of pertinent outcome measures, and studies not published in English.

### 2.2. Screening process

A meticulous 2-step screening process was employed to identify potential studies. During the initial screening, the titles and abstracts of identified articles were subjected to comprehensive review to gauge their relevance to the research topic. Subsequently, full-text articles that passed the initial screening were subjected to a meticulous evaluation vis-à-vis the predefined inclusion and exclusion criteria.

### 2.3. Data extraction

Relevant data were meticulously extracted from the selected studies. This encompassed critical information such as study design, sample size, key findings, and any acknowledged limitations.

### 2.4. Narrative synthesis

Given the inherent heterogeneity across the selected studies regarding design, outcomes, and the populations under investigation, a narrative synthesis approach was judiciously adopted. This facilitated summarizing findings from the selected studies and identifying common themes, emerging trends, and any notable disparities.

## 3. Cognitive domains affected by diabetes

Cognitive function represents diverse mental processes that empower individuals to perceive, analyze, store, and retrieve information from their surroundings. These cognitive processes orchestrate essential functions such as thinking, reasoning, solving problems, remembering, and effective communication. While cognitive function is often portrayed as a unified construct, it can be dissected into several distinct domains, each representing a specialized facet of mental processing. Understanding how diabetes intricately affects these cognitive domains is paramount for comprehending the full scope of its impact on individuals’ lives and for devising targeted interventions to mitigate cognitive decline.^[7,8]^

### 3.1. Memory

Memory, often considered one of the most pivotal cognitive domains, has been extensively scrutinized in the context of diabetes. Memory can be further fractionated into short-term, long-term, and working categories. A wealth of research has consistently unveiled a heightened risk of memory deficits in individuals with diabetes, especially those struggling to maintain adequate blood glucose control.^[9–11]^ These deficits can manifest as difficulties recalling recent events, names, or faces, profoundly affecting daily functioning and quality of life. The exact mechanisms behind these memory impairments are multifaceted and still under investigation. Still, they may involve the detrimental influence of chronic hyperglycemia on brain structures and neural networks crucial for memory processes.^[12]^

### 3.2. Attention and concentration

Among the cognitive functions, attention and concentration are pillars of mental prowess, enabling individuals to focus on specific tasks, filter out distractions, and sustain mental effort over extended periods.^[13]^ Diabetes-related cognitive impairments in these domains often manifest as heightened distractibility, diminished multitasking abilities, and difficulty maintaining sustained attention.^[14]^ These deficits can have practical ramifications on daily activities, impacting an individual capacity to perform tasks demanding prolonged focus, such as driving or workplace productivity.

### 3.3. Language

The domain of language, which underpins our capacity to comprehend and communicate using words and symbols, is indispensable to human interaction and expression.^[15]^ In diabetes, language deficits may emerge as difficulties finding the right words (anomia), impaired comprehension of complex sentences, or reduced fluency in spoken and written communication.^[16]^ These language impairments can result in communication challenges and have a tangible impact on an individual overall quality of life.

### 3.4. Executive function

Executive function, a higher-order cognitive domain, encompasses a wide spectrum of mental processes critical for planning, organization, problem-solving, decision-making, and impulse control.^[17]^ It plays an instrumental role in goal-directed behavior and is indispensable for effectively managing daily tasks. Diabetes has been associated with deficits in executive function, which can manifest as challenges in adhering to diabetes self-care regimens, including medication adherence and dietary choices.^[18]^ These impairments can initiate a domino effect on overall diabetes management and glycemic control.

### 3.5. Visuo-spatial skills

Visuo-spatial skills encompass the ability to perceive, analyze, and manipulate visual information in one environment.^[19]^ These skills are integral for reading maps, navigating unfamiliar surroundings, and engaging in spatial reasoning. Emerging research indicates that diabetes may influence visuospatial skills, potentially leading to spatial orientation and navigation difficulties, significantly impacting an individual autonomy and safety.^[20]^

## 4. Contributing factors to diabetes-related cognitive impairment

**Figure 1.**
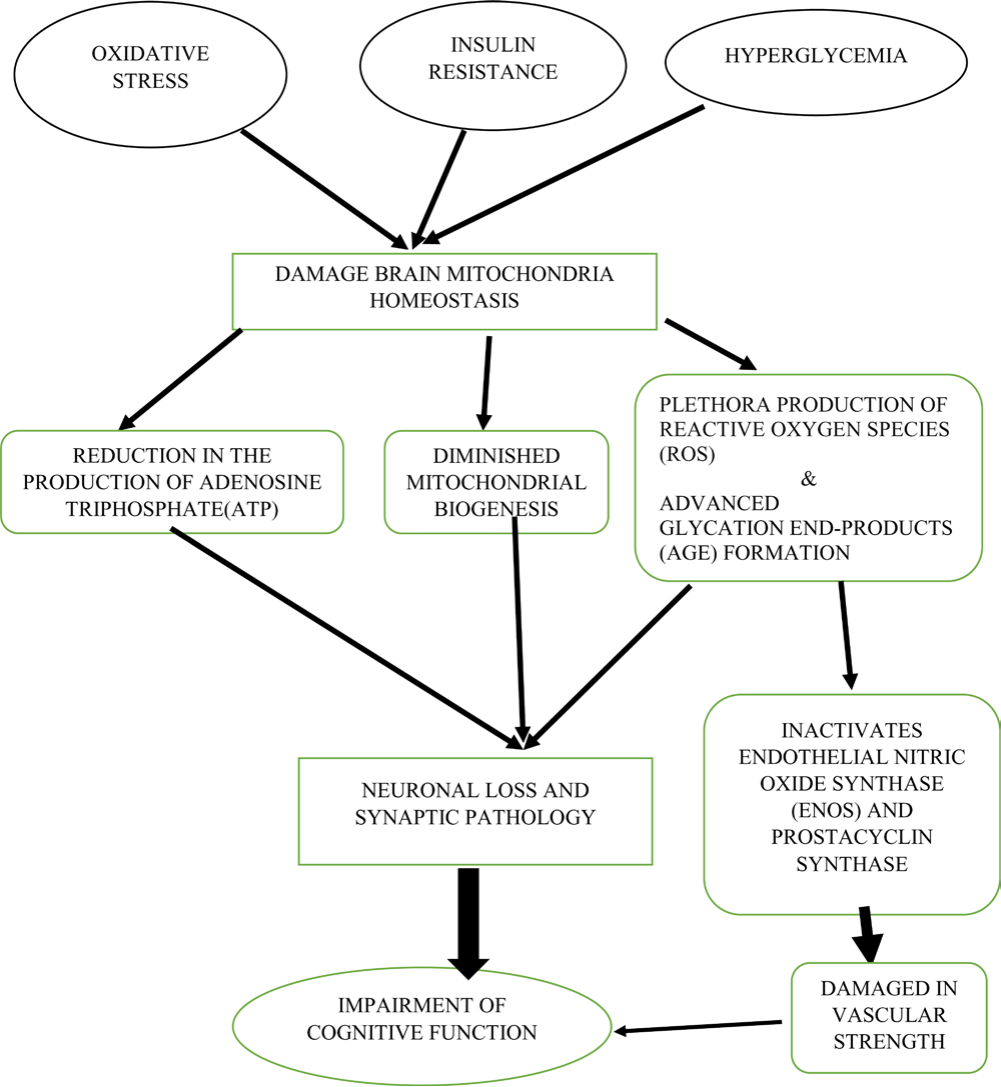
The potential pathways between diabetes-related factors and cognitive impairment.

Many contributing factors influence diabetes-related cognitive impairment, each intricately shaping the cognitive landscape of individuals with diabetes (Fig. 1).

### 4.1. Chronic hyperglycemia and glycemic variability

Elevated blood glucose levels, a hallmark of diabetes, stand as primary drivers of cognitive impairment.^[21]^ Prolonged hyperglycemia triggers a cascade of detrimental processes, including increased production of reactive oxygen species (ROS), notably superoxide.^[22]^ These ROS initiate adverse effects such as heightened polyol pathway activation, advanced glycation end products (AGEs) formation, protein kinase C activation, and intensified glucose shunting in the hexosamine pathway.^[22]^ These processes ultimately result in oxidative damage and vascular complications. Chronic hyperglycemia closely links to endothelial dysfunction, primarily via polyol pathway activation.^[23]^ This process depletes nicotinamide adenine dinucleotide phosphate, diminishing endothelial nitric oxide synthase activity and reducing nitric oxide production. Consequences extend to atherosclerosis, thrombus formation, and cerebral infarction, all contributing to cognitive impairment.^[24]^

### 4.2. Insulin resistance and dysregulation

Cognitive impairment in diabetes is closely tied to insulin resistance and dysregulation.^[25]^ Brain regions housing insulin receptors (IRs), including the hippocampus and frontal cortex, are essential for insulin cognitive effects.^[26]^ IRs are distributed throughout the brain, enabling insulin and insulin-like growth factor 1 to exert their biological influence.^[27]^ Insulin resistance and hyperinsulinemia, prevalent in diabetes, negatively affect amyloid processing and accumulation.^[27]^ This leads to increased intraneuronal β-amyloid deposition, tau hyperphosphorylation, and reduced β-amyloid clearance. Simultaneously, insulin resistance disrupts the blood-brain barrier, impacting cerebrovascular function and cognition.^[27]^ In addition, Insulin resistance is often accompanied by chronic low-grade inflammation, a state referred to as “meta-inflammation.^[26]^” This inflammatory condition can extend to the brain and contribute to neuroinflammation.^[26]^ Chronic neuroinflammation is associated with various cognitive disorders.^[27]^

### 4.3. Vascular complications and microvascular dysfunction

Cognitive impairment in diabetes is significantly influenced by its impact on microvascular and macrovascular systems.^[28]^ Hyperglycemia underpins microvascular complications, leading to diabetic nephropathy, neuropathy, and retinopathy. Macrovascular complications encompass cardiovascular and cerebrovascular diseases.^[29]^ The integrity of neurovascular units, responsible for regulating cerebral blood flow, is compromised in diabetes. Structural changes in microvasculature, including capillary reduction and arteriovenous shortcuts, affect nerve tissue nutrient delivery. This renders the brain more susceptible to oxygen shortages, potentially leading to cognitive impairment.^[30]^

### 4.4. Inflammation and oxidative stress

Oxidative stress and inflammation contribute significantly to diabetes-related cognitive impairment.^[31]^ Hyperglycemia promotes the generation of ROS and reactive nitrogen species, leading to oxidative damage across various biological pathways.^[31]^ Oxidative stress contributes to neuronal injury via osmotic insults and the accumulation of excitatory amino acids like glutamate. Additionally, AGEs activate microglia, the brain immune cells, potentially harming neurons.^[32]^ Individuals with diabetes often exhibit heightened inflammation characterized by increased inflammatory cytokine release, further exacerbating cognitive decline.^[32]^

### 4.5. Genetic and epigenetic factors

Genetic and epigenetic influences complicate the diabetes-cognition interplay. The APOE ε4 allele, associated with Alzheimer disease (AD), elevates the risk of cognitive impairment when combined with diabetes.^[33]^ The insulin-degrading enzyme (IDE) gene mediates amyloid-β and insulin breakdown, influencing cognitive outcomes.^[34]^

## 5. Mechanisms underlying the relationship between diabetes and cognitive impairment

**Figure 2.**
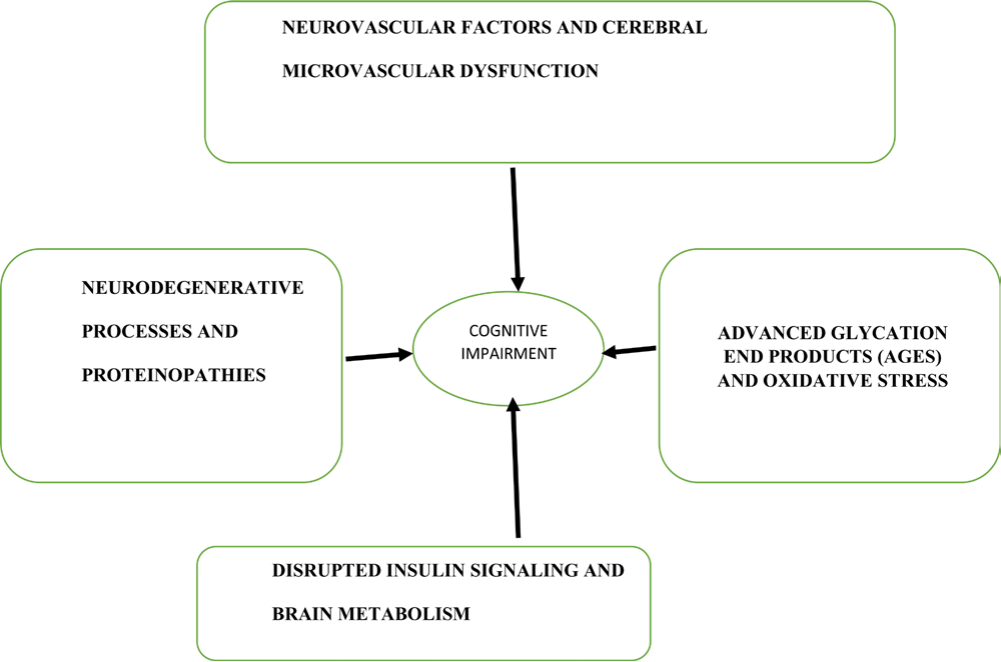
Mechanisms underlying diabetes-related cognitive impairment.

The etiology of cognitive impairment in individuals with diabetes is a complex and evolving topic.^[3]^ Emerging evidence suggests that altered blood-brain barrier (BBB) function, particularly associated with cerebral microvascular dysfunction, plays a pivotal role^[35]^ (Fig. 2).

### 5.1. Neurovascular factors and cerebral microvascular dysfunction

The BBB, composed of vascular endothelium in brain microvessels and adjacent astrocytic end-feet processes, regulates substance transport to and from the brain parenchyma.^[36]^ Tight endothelial junctions, minimal fenestration, and low pinocytic trafficking maintain the brain microenvironment.^[36]^ Astrocytes, essential for BBB regulation, release signals like transforming growth factor β and vascular endothelial growth factor, influencing BBB permeability and neurovascular coupling (NVC).^[37,38]^ Experimental evidence, though subject to debate, suggests that diabetes compromises BBB integrity, increasing barrier permeability.^[36]^ Prolonged hyperglycemia also impairs astrocytic gap junctional communication, potentially disrupting NVC and contributing to cognitive impairment.^[39]^ Reduced nitric oxide availability, often due to oxidative stress from AGEs, may further impair NVC in diabetes mellitus, potentially linking to cognitive decline.^[40]^

### 5.2. Neurodegenerative processes and proteinopathies

Neurodegenerative diseases like AD, Huntington disease, and Parkinson disease are characterized by protein aggregation, termed proteinopathies.^[41]^ Abnormal protein aggregates, primarily amyloids, result from protein misfolding and aggregation.^[42]^ Post-translational modifications and protein-quality control mechanisms contribute to misfolded protein aggregation.^[43,44,45]^ Accumulation of misfolded oligomers in the endoplasmic reticulum (ER) induces ER stress, releasing inflammatory mediators and initiating apoptotic pathways, ultimately causing neurotoxicity.^[46]^ Protein aggregation and neuroinflammation are hallmarks of neurodegenerative diseases.^[46]^

### 5.3. Disrupted insulin signaling and brain metabolism

Insulin, crossing the BBB, plays a crucial role in cognitive processes and food intake regulation.^[47]^ IRs in regions like the hippocampus and frontal cortex impact memory and neurotransmitter regulation.^[48]^ Insulin resistance disrupts these processes.^[48]^ Hyperinsulinemia, common in insulin resistance, decreases BBB permeability, limiting brain insulin entry.^[49]^ Reduced insulin signaling affects AD pathology by influencing tau phosphorylation and amyloid-beta metabolism, promoting neurofibrillary tangle formation and amyloid accumulation.^[49]^ It also reduces IDE levels, potentially increasing amyloid accumulation.^[50]^

### 5.4. Advanced glycation end products (AGEs) and oxidative stress

AGEs, formed through non-enzymatic glycation of proteins or lipids exposed to aldose sugars, elevated oxidative stress and inflammation.^[51,52]^ This leads to increased amyloid beta-42 (Aβ 42) production. Microglial clearance of Aβ 42, hindered in diabetes, results in greater Aβ accumulation and inflammation.^[53]^ RAGE and Aβ expression are elevated in specific brain regions of diabetic rats.^[54]^

## 6. Studies investigating the role of diabetes in cognitive impairment

The investigation into the intricate relationship between diabetes and cognitive impairment has garnered substantial attention in recent years (Table 2). As diabetes mellitus continues its global rise, extending beyond its conventional implications on metabolic health, researchers have increasingly focused on its potential impact on cognitive function.

**Table 2:** Studies investigating the role of diabetes in cognitive impairment.

Study author & yr	Participants	Duration	Cognitive tests	Main findings
Palta P. et al (2017)	3069 adults aged 72–96 yr	Median follow-up of 6.1 yr	Memory, visuo-spatial construction, language, psychomotor speed, executive function	Older adults with diabetes exhibited greater baseline differences in executive function and greater declines in language. No significant differences in the rate of cognitive decline in composite cognitive domain scores.
Mallorquí-Bagué N. et al (2018)	6823 older individuals with overweight/obesity and metabolic syndrome	Cross-sectional analysis	Executive function and BMI were negatively associated with type 2 diabetes. Participants with type 2 diabetes and better glycemic control displayed better cognitive performance.	Type 2 diabetes was associated with worse executive function, and BMI had a negative effect on executive function.
Jacobson A.M. et al (2021)	1051 participants with type 1 diabetes	32 yr of follow-up	Memory, psychomotor and mental efficiency	Cognitive performance declined over 32 yr of follow-up. Higher HbA1c levels, severe hypoglycemia, and elevated systolic blood pressure were associated with greater cognitive decline.
Lehtisalo J. et al (2016)	364 participants with impaired glucose tolerance	Intervention period 4 yr	Cognitive assessment (CERAD test battery, Trail Making Test A)	Better glycemic control predicted better cognitive performance 9 yr later. Learning effects in cognitive testing were not evident in people with long diabetes duration.
Lutski M. et al (2017)	489 patients with cardiovascular disease	2 decades of follow-up	Cognitive function assessment	Insulin resistance was related to poorer cognitive performance and greater cognitive decline among patients with cardiovascular disease.
Hayden K.M. et al (2021)	3938 participants with type 2 diabetes	Up to 18 yr of follow-up	Cognitive function assessment	Intensive lifestyle intervention did not result in preserved cognitive function or slower rates of cognitive decline.
Espeland M.A. et al (2018)	3802 individuals with type 2 diabetes	10–13 yr of follow-up	Cognitive impairment assessment	Cognitive impairment prevalence was lower in women than in men among overweight and obese adults with type 2 diabetes.
Williamson J.D. et al (2014)	2977 participants with type 2 diabetes	40 mo of follow-up	Cognitive function and total brain volume assessment	Intensive blood pressure control was associated with greater decline in total brain volume.
An K. et al (2021)	235 Patients with type 2 diabetes	Cognitive assessment	Verbal disfluency and cognitive performance	Decreased plasma level of lipoprotein lipase predicted early cognitive deficits.
Furlano J.A. et al (2023)	124 60–80 yr old with prediabetes or overweight/obesity	6 mo of resistance training	Cognitive ability assessment, functional MRI	Resistance training improved cognitive ability and functional MRI patterns.
Emanuel A.L. et al (2019)	25 type 1 diabetes patients with retinopathy	Longitudinal study	Cognitive performance, cerebral blood flow (CBF)	Lower cognitive performance was associated with white matter lesions and lower skin capillary perfusion in type 1 diabetes patients.
Cukierman-Yaffe T. et al (2015)	31,227 participants with diabetes	Median follow-up of 4.7 yr	Relationship between fasting plasma glucose values and dementia, cognitive decline, and cognitive impairment	Higher fasting plasma glucose values were associated with an increased risk of dementia, cognitive decline, and cognitive impairment in people with diabetes.
Lotan R. et al (2021)	544 Older adults with type 2 diabetes	6 mo of dietary AGEs reduction	Cognitive performance, olfactory function, odor-induced brain alterations	Dietary AGEs reduction improved cognition and olfactory function in obese individuals with type 2 diabetes.
Zhang Z. et al (2019)	800 Obese and nonobese people with type 2 diabetes	Cross-sectional and 3-mo intervention	Cognitive functioning, olfactory function, brain activation	Obese individuals with type 2 diabetes had worse cognitive function and olfactory function, which improved with weight loss and improved glycemic control.
Spauwen P.J.J., et al (2013)	704 participants with type 2 diabetes	5 yr of follow-up	Cognitive function assessment	Presence of cerebral small vessel disease was associated with cognitive decline in type 2 diabetes.
Zheng F., Yan L., Yang Z. et al (2018)	5189	10 yr	Global cognitive z scores, memory z scores, executive function z scores	- 1 mmol/mol increment in HbA1c associated with cognitive decline < br> - Prediabetes and diabetes linked to increased cognitive decline
Botond Antal et al (2022)	20,314	Not specified	Not specified	- T2DM associated with cognitive deficits, especially executive function < br> - Gray matter atrophy in T2DM < br> - Metformin didn’t improve outcomes
Moran C. et al (2019)	705	4.6 yr	Verbal memory, cortical thickness	- T2DM associated with decline in verbal memory and fluency < br> - T2DM linked to lower baseline cortical thickness
Xie K. et al (2022)	732	5–8 yr	COGTEL scores, memory, working memory	- Lower cognitive performance in T2DM < br> - Memory-related domains sensitive to T2DM
Callisaya M.L. et al (2019)	Not specified	5 yr	Cortical thickness, cognitive function	- T2DM associated with cognitive decline via neurodegeneration
Varghese S.M. et al (2022)	800	Not specified	Addenbrooke Cognitive Examination-III	- Cognitive impairment in 63.8% of diabetics < br> - Memory-related domains affected
Rawlings A.M. et al (2014)	13,351	20 yr	Cognitive function	- Diabetes linked to greater cognitive decline < br> - Prediabetes associated with cognitive decline
Lalithambika C.V. et al (2019)	70	Not specified	Montreal Cognitive Assessment (MoCA)	- High prevalence of MCI in type 2 diabetic patients < br> - Poor glucose control correlated with cognitive impairment
Bashir J. et al (2022)	61	Not specified	Montreal Cognitive Assessment test (MoCA)	- MCI common in advanced T2DM < br> - Hyperinsulinaemia correlated with MCI
Crane P.K. et al (2013)	2067	6.8 yr	Clinical measurements of glucose levels	- Higher glucose levels associated with increased dementia risk in non-diabetic and diabetic individuals
Hazari M.A.H. et al (2015)	46	Not specified	P300 event-related potentials (ERPs)	- Cognitive dysfunction in T2DM, more pronounced with longer disease duration < br> - Hypertension worsened cognitive function

AGEs = advanced glycation end products.

### 6.1. Executive function and cognitive decline

Several studies, including Palta et al (2017), Mallorquí-Bagué et al (2018), and Jacobson et al (2021), consistently point to pronounced vulnerabilities in executive function among older adults with diabetes.^[55–57]^ The consistent findings across these studies emphasize the pivotal role of executive function in diabetes-related cognitive deterioration. This cognitive domain is essential for making choices, managing daily activities, and adapting to new situations. Planning, organizing, and controlling impulses are vital for maintaining independence and overall quality of life. Therefore, the observed vulnerabilities in executive function among individuals with diabetes significantly affect their daily functioning and well-being.

### 6.2. Glycemic control

Maintaining optimal glycemic control emerges as a cornerstone in preserving cognitive function. Lehtisalo et al (2016) and Mallorquí-Bagué et al (2018) emphasize the significance of glycemic control in predicting improved cognitive performance.^[56,58]^ Superior glycemic control is associated with better cognitive outcomes, highlighting the pivotal role of blood sugar regulation in cognitive health.

### 6.3. BMI and obesity

The negative association between type 2 diabetes and executive function is exacerbated by higher BMI, as observed in the study by Mallorquí-Bagué et al (2018).^[56]^ Additionally, Zhang et al (2019) demonstrate that obesity in individuals with type 2 diabetes worsens cognitive function.^[59]^ Weight loss and improved glycemic control can ameliorate these cognitive deficits, underscoring the adverse impact of obesity on cognitive function in diabetes.

### 6.4. Neurodegeneration

Studies by Botond Antal et al (2022) and Callisaya ML et al (2019) provide evidence that diabetes contributes to cognitive decline through neurodegenerative processes.^[60,61]^ Structural brain changes, including gray matter atrophy and cortical thickness alterations, point to the intricate relationship between diabetes, neurodegeneration, and cognitive impairment.

### 6.5. Lifestyle interventions

The effectiveness of lifestyle interventions in preserving cognitive function remains debated. Hayden et al (2021) found that intensive lifestyle intervention did not consistently lead to preserved cognitive function or reduced cognitive decline in type 2 diabetes patients.^[62]^ This raises questions about the efficacy and individual variability in response to such interventions in the context of cognitive outcomes.

### 6.6. Gender differences

Gender disparities in cognitive impairment prevalence among overweight and obese adults with type 2 diabetes are highlighted by Espeland et al (2018).^[63]^ Their study reports lower cognitive impairment rates in women than men in this demographic, suggesting potential gender-related variations in cognitive outcomes.

### 6.7. Vascular factors

Multiple studies (Spauwen et al, 2013; Cukierman-Yaffe et al, 2015; Crane PK et al, 2013) emphasize the influence of vascular factors on cognitive function.^[64–66]^ These factors include cerebral small vessel disease, glycemic control, and glucose levels, which are associated with an increased risk of dementia and cognitive decline. This relationship extends to diabetic and non-diabetic individuals, emphasizing the significance of vascular health.

### 6.8. Dietary modifications

Lotan et al (2021) provide evidence that dietary modifications, specifically reducing advanced glycation end products (AGEs), can improve cognition and olfactory function in individuals with type 2 diabetes.^[67]^ This suggests the potential benefits of dietary interventions in preserving cognitive health.

## 7. Clinical implications and consequences of diabetes-related cognitive impairment

The growing body of evidence highlighting the pathological interplay between metabolic dysfunction, such as diabetes mellitus, and susceptibility to cognitive impairment is of significant clinical concern.^[68]^ Previous research has firmly established an association between diabetes and progressive cognitive decline, spanning from diabetes-related cognitive deficits to mild cognitive impairment (MCI), both non-amnesic and amnesic, and eventually to dementia, including vascular and Alzheimer dementia.^[69]^

### 7.1. Impact on daily functioning, disease management, and quality of life

Diabetes mellitus is closely intertwined with diminished neurocognitive function, driven by various mechanisms, including vascular diseases and defects in insulin metabolism, which can lead to the deposition of amyloid-β in the central nervous system.^[70]^ The use of insulin therapy, while crucial in diabetes management, can have unintended consequences such as decreased IDE production, fostering the formation of amyloid plaques and glycated end products associated with dementia, particularly AD.^[71]^ Notably, diabetes often coexists with depression, a comorbidity associated with dementia. Diabetic individuals with comorbid depression face a 2.7-fold increased risk of dementia compared to those with diabetes alone.^[72]^ Furthermore, diabetes established link with heightened cardiovascular risk and micro- and macrovascular cerebral diseases further contributes to a diminished quality of life.^[73]^ Concerns have been raised regarding the use of DPP4 inhibitors, a common treatment for diabetes with a relatively lower incidence of dementia, concerning heart failure and other cardiovascular complications.^[74]^ However, the potential predisposition of patients to heart failure or cardiovascular issues due to DPP4 inhibitors remains a topic of debate, necessitating future studies for clarification.^[75]^ Cognitive impairment in diabetics extends to various domains, often resulting in reduced performance in attention and executive functions, information processing, and memory.^[76]^

### 7.2. Challenges in diagnosis, screening, and management of cognitive impairment in diabetes patients

Diagnosing cognitive decline in diabetic patients presents significant challenges, especially in the mild cognitive impairment stage. Early diagnosis is further complicated by patients’ reluctance to seek healthcare. The American Diabetes Association has recommended screening for cognitive impairment in individuals with type 2 diabetes aged 65 and above during the initial visit and annually after that.^[77]^ Given the disease burden and the complexities of managing advanced dementia in these patients, early screening is crucial, especially considering the presence of microvascular and macrovascular complications and fluctuating glycemic states, predisposing them to cognitive dysfunction.^[78]^ The American Diabetes Association guidelines currently suggest employing cognitive screening tools such as the Mini-Mental State Examination (MMSE) and the Montreal Cognitive Assessment (MoCA) when dementia is suspected.^[79]^ However, these tools have their limitations. A comprehensive evaluation should encompass various cognitive domains, including abstract reasoning, information processing speed, attention and executive function, memory (including working memory, immediate memory, learning rate, forgetting rate, and incidental memory), and visuospatial skills.^[80]^ While the MMSE has been widely used, its effectiveness in diagnosing dementia in early cognitive impairment remains questionable, with limited diagnostic utility.^[81]^ The MoCA is a more reliable screening tool than the MMSE but has its limitations, including the need for a trained expert to administer it and susceptibility to patient literacy and mood.^[82]^ Emerging methods for screening and monitoring cognitive impairment in type 2 diabetic patients over 65 have shown promise. The Diabetes Specific Dementia Risk Score (DSDRS) and retinal microperimetry have demonstrated increased sensitivity and specificity compared to traditional MMSE and MoCA scores.^[83]^ The DSDRS, originally designed as a risk score for predicting 10-year dementia risk in type 2 diabetic patients, has proven to be a reliable screening tool. The European Consortium on Models of Patient Engagement for Alzheimer Disease project (MOPEAD) used the DSDRS to screen for cognitive impairment in type 2 diabetics, revealing a higher prevalence of undiagnosed cognitive dysfunction in this population. Neuropsychological tests further confirmed the utility of the DSDRS as a tool for diagnosing mild cognitive impairment.^[84]^ Retinal microperimetry, a noninvasive test measuring light intensity in the eye when light strikes the retina fovea, also shows promise. Its high sensitivity correlates with imaging findings in MCI and dementia, making it a valuable tool for screening and monitoring cognitive impairment in type 2 diabetic patients.^[85]^

### 7.3. Interdisciplinary approaches and integrated care considerations

Managing diabetes-related cognitive decline necessitates a multidisciplinary approach involving diabetologists/endocrinologists, dieticians, neurologists, cardiologists, dementia specialists, primary healthcare physicians, psychiatrists, geriatricians, specialized nurses, and physical therapists.^[86]^ Bridging the gaps between neuroscientific subfields and basic biomedical and clinical sciences is vital for providing optimal care to patients with diabetes and cognitive dysfunction. Psychiatrists and primary care physicians play essential roles in assessing additional risk factors and comorbidities in diabetic individuals with cognitive impairment, involving specialists when necessary.^[87]^ Given the frequent coexistence of cardiovascular and metabolic conditions, including hypertension, dyslipidemia, weight gain, and metabolic syndrome among these patients, cardiologists and endocrinologists are essential in ensuring comprehensive cardiovascular risk assessments and subsequent diagnostic workups for improved diagnosis and management.^[88]^

## 8. Interventions and management strategies

In pursuing effective interventions for controlling hyperglycemia and mitigating the underlying pathology of cognitive decline in diabetes, there is a growing need and advocacy for developing new and more potent therapies to reduce and attenuate cognitive deficits in diabetic individuals.^[89]^ One pivotal strategy involves targeting the neuroinflammation resulting from hyperglycemia-induced overproduction of ROS and inflammatory cytokines.^[90]^ Recent antidiabetic agents, such as glucagon-like peptide-1 receptor agonists (GLP-1RAs), have shown promise in managing hyperglycemia and addressing the underlying pathology of cognitive dysfunction.

### 8.1. Lifestyle modifications for cognitive preservation

Elevated levels of islet amyloid polypeptide in diabetic individuals are linked to insulin resistance and cognitive decline.^[91]^ Lifestyle changes can play a pivotal role in preserving cognitive function. Exercise has been shown to improve cognitive function and correct existing dysfunction. For example, a 6-month aerobic exercise program improved executive function in individuals with type 2 diabetes, impaired fasting glucose, and impaired glucose tolerance.^[92]^ Dietary modifications, such as adopting a Mediterranean diet, have been associated with better glycemic control and cognitive preservation.^[93]^ Research suggests that adherence to this diet is linked to improved cognitive function and a lower risk of cognitive impairment.^[94]^

### 8.2. Pharmacological interventions targeting cognitive impairment

Numerous pharmacotherapeutic agents are employed in managing diabetes and reducing the risk of cognitive impairment, including dementia. These agents belong to various classes of oral hypoglycemic agents, such as biguanides, sulfonylureas, thiazolidinediones, dipeptidyl peptidase-4 inhibitors, and glucagon-like peptide-1 (GLP-1) agonists.^[95]^ Recent studies have highlighted the protective effects of DPP-4 inhibitors, particularly in combination with metformin, on reducing dementia risk.^[96,97]^ Similarly, sodium-glucose cotransporter 2 inhibitors significantly reduce cognitive dysfunction.^[98]^ Other pharmacological agents, including Phenibut and Ipidacrine, have been explored as additional therapies for diabetic patients.^[99]^

### 8.3. Optimization of glycemic control and its impact on cognitive outcomes

Glycemic control is pivotal in mitigating cognitive impairment in diabetes. Several studies have indicated a direct link between poor glycemic control and cognitive decline, including the onset of dementia.^[56,57]^ Controlling blood sugar levels is crucial in preventing the deterioration of cognitive function. For instance, high glycated hemoglobin A1c concentrations are associated with a progressive decline in cognitive function, particularly in memory and executive function.^[100]^ However, it is worth noting that insulin, while effective for glycemic control, has been associated with diabetes-induced neurocognitive decline.^[101]^ Intranasal insulin therapy shows promise in improving memory function.^[101]^ Although intensive glycemic control has been linked to reduced brain atrophy, no conclusive evidence supports its superiority over standard control.^[102]^

### 8.4. Potential therapeutic advancements and novel interventions

Novel therapies are emerging to preserve cognitive function while minimizing side effects in diabetics.^[103]^ These therapies target various pathways, including incretins like glucose-dependent insulinotropic polypeptide (GIP) and glucagon-like peptide 1 (GLP-1).^[104]^ Longer-acting GLP-1 receptor agonists (GLP-1RAs) in combination with DPP-4 inhibitors show potential for preserving cognitive function and mitigating brain damage resulting from high-fat diets.^[105]^ Dual incretin agonists stimulating GLP-1 and GIP receptors have shown neuroprotective effects, reducing markers of neuroinflammation and neurodegeneration.^[106]^ Triple agonists that stimulate GLP-1R, GIPR, and glucagon receptor (GcgR) demonstrate metabolic effects and potency in reducing neuroinflammation and neurodegeneration.^[107]^ Additionally, cognitive training may slow cognitive decline in elderly patients, although further research is needed. Ongoing clinical trials are investigating the effects of different antidiabetic agents on cognitive decline.^[108]^ Agents like Liraglutide and Lixisenatide show the potential to reverse memory impairment.^[108]^ In clinical trials, GLP-1 analogues and other antidiabetic medications have demonstrated therapeutic effects in managing neurocognitive decline.^[109,110]^ Furthermore, antioxidants and anti-inflammatory agents, including Sesamol, curcumin, and vitamin E, have improved cognitive functions in rats.^[110]^

## 9. Future directions and research prospects

### 9.1. Identifying research gaps and limitations in the current evidence

Despite the significant body of research in this area, several research gaps and limitations remain. One major drawback is the lack of consistency in defining cognitive impairment and its assessment across studies. The absence of a widely agreed-upon diagnostic paradigm hinders the ability to compare and generalize results. Furthermore, most current research focuses on elderly individuals with diabetes, overlooking the implications of cognitive impairment in younger populations and its potential long-term consequences.^[111]^ Moreover, there is a relative scarcity of evidence concerning the potential impacts of other types of diabetes, as most studies have predominantly explored the relationship between type 2 diabetes and cognitive impairment. This underscores the need for more research into how type 1 diabetes and gestational diabetes may affect cognitive function. Additionally, there is a need for further investigation into the effects of diabetes-related comorbidities, such as hypertension and dyslipidemia, which may act in concert to influence cognitive decline. Most available evidence is cross-sectional, limiting our ability to establish causal relationships. Longitudinal studies are essential for understanding disease progression, identifying risk factors, and establishing temporal correlations between diabetes and cognitive impairment. Furthermore, current research often overlooks the impact of lifestyle changes, treatment regimens, and diabetes management on cognitive outcomes. To develop effective prevention and intervention strategies, examining the effects of various therapeutic modalities and lifestyle modifications on cognitive performance is crucial.

### 9.2. Need for longitudinal studies and mechanistic investigations

Future research should prioritize longitudinal studies to overcome the limitations of cross-sectional studies and gain a deeper understanding of the complex interactions between diabetes and cognitive impairment. Long-term observational studies can track changes in cognition over time and help determine whether diabetes acts as an independent risk factor for cognitive decline or merely as a contributing component. Findings from such research may also identify critical windows of vulnerability during diabetes, indicating optimal times for preventive interventions. Furthermore, interdisciplinary research is needed to elucidate the molecular underpinnings of cognitive impairment in diabetes. Integrating neuroscience, endocrinology, and immunology may offer insights into the neurobiological pathways linking diabetes and cognitive decline. Mechanistic investigations could explore the roles of persistent hyperglycemia, insulin resistance, synapse loss, and neuroinflammation.

### 9.3. Potential biomarkers and predictive factors for cognitive impairment in diabetes

Efforts should be directed toward identifying accurate biomarkers and predictive indicators for cognitive impairment in individuals with diabetes. Exploring potential biomarkers in blood, cerebrospinal fluid, or neuroimaging that correlate with cognitive decline in diabetes should be a primary focus of future research. Biomarkers related to glucose metabolism, oxidative stress, and neuroinflammation may provide crucial diagnostic and prognostic information. Additionally, genetic and epigenetic factors should be incorporated into the search for predictive markers alongside traditional clinical criteria. Understanding how individual genetic variations or epigenetic changes influence cognitive outcomes may aid in risk assessment and developing personalized treatment modalities.

### 9.4. Personalized approaches and tailored interventions

Given the significant variability in cognitive outcomes among people with diabetes, personalized strategies to prevent or manage cognitive impairment are essential. Individualized approaches should consider variations in diabetes duration, glycemic control, comorbidities, and lifestyle factors when tailoring therapies. Depending on an individual diabetic profile, lifestyle interventions such as cognitive training, physical activity, and dietary modifications may affect cognitive performance. Thus, determining the optimal combination of interventions for specific populations is crucial. In tailored therapy, wearables and mobile health applications may facilitate remote monitoring and enhance patient compliance. Furthermore, pharmacogenetic features in the interplay between diabetes and cognitive impairment are an important and evolving area of research. The APOE ε4 allele has long been recognized as a significant genetic risk factor for AD.^[29]^ For example, when individuals with diabetes carry this allele, it appears to elevate their risk of cognitive impairment even further. This suggests a synergistic effect between diabetes-related metabolic disruptions and genetic susceptibility. Research in this area is vital for identifying individuals at the highest risk for cognitive decline and tailoring interventions accordingly.

## 10. Limitations and strengths of review

In our earnest pursuit of conducting a comprehensive review, it is important to acknowledge certain limitations that may have influenced the scope and findings of this analysis. The time frame of the included studies bounds our review. As such, older studies may have surfaced after this review, which could hold significant relevance to the topic. Conversely, this review benefits from a series of notable strengths. Our commitment to conducting a comprehensive literature search involved systematically exploring multiple databases and sources. This approach ensured a thorough and inclusive collection of evidence.

## 11. Conclusion

The impact of diabetes on cognitive function is a complex and multifaceted issue that requires comprehensive understanding and targeted interventions. As we have explored the various cognitive domains affected by diabetes, the contributing factors, underlying mechanisms, and the findings of research studies, it becomes evident that cognitive impairment is a significant concern for individuals with diabetes. Memory deficits, attention and concentration challenges, language difficulties, executive function impairments, and visuospatial skills disruptions all underscore the far-reaching consequences of this condition. The contributing factors, such as chronic hyperglycemia, insulin resistance, vascular complications, inflammation, and genetic influences, add complexity to this relationship. Understanding these factors is essential for developing effective prevention and intervention strategies. Studies examining the role of diabetes in cognitive decline have consistently pointed to vulnerabilities in executive function, the significance of glycemic control, the impact of obesity, neurodegenerative processes, and the potential benefits of dietary modifications and lifestyle interventions. However, diagnosis, screening, and management challenges persist, highlighting the need for improved tools and early screening practices.

Clinical implications are profound, affecting daily functioning, disease management, and overall quality of life for individuals with diabetes. The interdisciplinary approach to care is vital, with various medical specialists working together to provide comprehensive support and management. Interventions and management strategies encompass lifestyle modifications, pharmacological options, and optimizing glycemic control. Promising novel therapies are emerging, targeting neuroinflammation and neurodegenerative processes. Future research should prioritize longitudinal studies, mechanistic investigations, and the search for biomarkers and predictive factors. Personalized approaches and tailored interventions are the way forward, recognizing the individual variability in diabetes-related cognitive decline.

## Author contributions

**Conceptualization:** Nicholas Aderinto, Gbolahan Olatunji.

**Writing – original draft:** Nicholas Aderinto, Gbolahan Olatunji, Muili Abdulbasit, Patrick Ashinze, Olamide Faturoti, Abayomi Ajagbe, Bonaventure Ukoaka, Gbolahan Aboderin.

**Writing – review & editing:** Nicholas Aderinto, Gbolahan Olatunji, Muili Abdulbasit, Patrick Ashinze, Olamide Faturoti, Abayomi Ajagbe, Bonaventure Ukoaka, Gbolahan Aboderin.
